# Effects of overwintering on the transcriptome and fitness traits in a damselfly with variable voltinism across two latitudes

**DOI:** 10.1038/s41598-024-63066-z

**Published:** 2024-05-28

**Authors:** Guillaume Wos, Gemma Palomar, Maria J. Golab, Marzena Marszałek, Szymon Sniegula

**Affiliations:** 1 Institute of Nature Conservation Polish Academy of Sciences, Al. Adama Mickiewicza 33, 31-120 Kraków, Poland; 2 Department of Genetics, Physiology and Microbiology, Faculty of Biological Sciences, Complutense University of Madrid, José Antonio Novais, 12, 28040 Madrid, Spain; 3 Institute of Environmental Sciences, Jagiellonian University, Gronostajowa 7, 30-387 Kraków, Poland

**Keywords:** Ecological genetics, Evolutionary ecology, Evolutionary developmental biology, Population genetics, Entomology, Development

## Abstract

Winter diapause consists of cessation of development that allows individuals to survive unfavourable conditions. Winter diapause may bear various costs and questions have been raised about the evolutionary mechanisms maintaining facultative diapause. Here, we explored to what extent a facultative winter diapause affects life-history traits and the transcriptome in the damselfly *Ischnura elegans,* and whether these effects were latitude-specific. We collected adult females at central and high latitudes and raised their larvae in growth chambers. Larvae were split into a non-diapausing and post-winter (diapausing) cohort, were phenotyped and collected for a gene expression analysis. At the phenotypic level, we found no difference in survival between the two cohorts, and the post-winter cohort was larger and heavier than the non-winter cohort. These effects were mostly independent of the latitude of origin. At the transcriptomic level, wintering affected gene expression with a small fraction of genes significantly overlapping across latitudes, especially those related to morphogenesis. In conclusion, we found clear effects of diapause on the phenotype but little evidence for latitudinal-specific effects of diapause. Our results showed a shared transcriptomic basis underpinning diapause demonstrated, here, at the intraspecific level and supported the idea of evolutionary convergence of the response to diapause across organisms.

## Introduction

Diapause is a state consisting of cessation of development and reproduction that allows organisms to survive unfavourable conditions and/or to ensure their synchronization with favourable conditions^1,2^. Diapause has been studied in ectotherms especially in the context of winter diapause which is particularly important in temperate species as it is a crucial component of their life cycle^2,3^. Although in some insect species winter diapause is obligatory to terminate the development (one generation per year), in others it has also been shown to be facultative with coexisting diapausing and non-diapausing individuals (variable number of generations per year)^4^. As it has been assumed that winter diapause may bear various costs, i.e. metabolic or reproductive^5,6^, questions have been raised about the evolutionary mechanisms maintaining facultative diapause^7,8^. Hence, to gain insight into the role of a facultative winter diapause on organisms, a thorough comparison of diapausing and non-diapausing individuals on a set of life-history traits^9,10^ down to the gene level^11,12^ is necessary. Diapause is a complex state of developmental arrest which is controlled by hormones and by the environmental conditions^11^. Generally, in species with an obligate winter diapause, the contribution of the environment is reduced as diapausing is an integral part of organisms’ ontogeny. In contrast, in species with a facultative winter diapause, where two physiological alternatives exist, the contribution of the environment is more important^1^ and it also has a genetic basis^11^.

The latitudinal origin has often been invoked to explain differences in the presence or absence of diapause as latitude strongly correlates with temperature and photoperiod – two variables that trigger diapause^11^. Variation in temperature affects the developmental and growth rate, and in combination with seasonally changing photoperiod, the propensity to enter diapause in insects, i.e. higher propensity to enter larval diapause at higher latitudes^13^. This suggests that diapause dynamics are not necessarily uniform across a species range. In addition, the propensity to enter winter diapause may also vary between geographically close populations or even within a population, i.e. in beetles^14^ or in damselflies^15,16^. Such a difference in diapause at a local scale may arise from cohort splits defined as individuals from the same cohort (born at the same time) following different developmental trajectories and emerging in different years^16,17^. Factors inducing a cohort split are not fully understood but it has been proposed that individuals reaching a specific size (critical size) before a specific time of the year (critical time) might develop rapidly and emerge within the same growth season while the remaining individuals might delay development and emerge the following season^4,16,17^.

A number of empirical studies have investigated the effects of a facultative winter diapause on life-history traits^5,10,18,19^. In general, winter diapause may incur some costs as it correlates with some life-history traits which may impact survival and fecundity. For instance, diapausing individuals have been found to have lower mass and lipid reserves or lower fecundity compared to non-diapausing individuals in various insect species^5,10,18,19^. Winter diapause may also affect survival, with a decreased survival in diapausing individuals compared to non-diapausing ones^20^. As mentioned, variation in diapause dynamics may vary across a species’ range. For example, studies on the blow fly provided evidence that low- and high-latitude populations had different sensitivities to environmental cues (i.e. photoperiod) which induces larval winter diapause^19,21^. However, more studies are needed in order to assess differences in winter diapause across populations and its genetic underpinning.

While many studies have addressed changes in life-history traits, the knowledge about the genetic basis associated with diapause have received less attention. For example, during the induction of diapause, it was demonstrated changes in hormonal and neuronal pathways in butterflies^22^. Other studies tracked changes in gene expression over time during the winter period and revealed pathways related to protein metabolism and morphogenesis in apple maggot flies^23^ or compared gene expression patterns before and during diapause and showed differences in carbohydrate or catabolic processes in spider mites^24^. However, the integration of a geographic component in gene expression analyses to consider the variation in diapause dynamics across different populations has rarely been done^25^.

Here, we aimed at studying facultative winter diapause on the damselfly *Ischnura elegans* by comparing phenotype and transcriptomic profiles of individuals reaching the penultimate larval stage before and after a winter diapause period (non- vs post-winter cohort) and investigated similarities in gene expression patterns across two latitudes. We ran a common-garden experiment using larvae obtained from field-collected females from replicated high- (southern Sweden) and central-latitude (southern Poland) populations. We measured, in the non- and post-winter cohort, key larval life-history traits related to development, growth and body size, and collected these same individuals for a gene expression analysis by RNA-seq. We specifically asked (1) Do we observe differences in a set of life history traits between non-diapausing and diapausing cohorts? (2) Are these phenotypic differences between the two cohorts latitude-specific? (3) What are the genes differentially expressed between non- and post-wintering cohorts and (4) to what extent the differentially expressed genes overlap across the two latitudes?

## Methods

### Study species and collection

*Ischnura elegans* is a common damselfly in Europe, occurring from northern-central Sweden and Finland to southern Italy, Spain and Greece^26^. The damselfly has variable voltinism, asynchronous emergence and a breeding period that happens during spring and summer. High-latitude populations are uni- or semivoltine, i.e. one or two years for completing one generation, respectively. However, a non-overwintering generation, i.e. bivoltine cohort, has also been reported during a warm summer at high latitudes^16^ (Erik Svensson pers. comm.). Central-latitude populations are uni- and bivoltine, i.e. one or two generations per year^16,27^. Larvae show large variation in size throughout the growth season and during wintering^16,28^ with larval head width values, a proxy for larval structural body size and instar, to about 3.5 mm, which corresponds to the final instar prior emergence. Although overwintering was reported at final minus one (F-1) and final instar (F-0) before emergence in northern and central Europe^16^, in univoltine individuals there is a tendency for overwintering in final minus two (F-2) or earlier instars (head width < 2.5 mm)^29^. In semivoltine individuals (only in northern Europe), individuals overwinter at F-4 or earlier instars for the first winter and at F-1 and F-0 for the second winter^29^. Preliminary data suggest that shortening the photoperiod during the second part of the growth season in south Sweden populations induces winter diapause in F-2, F-1 and F-0 (no data for instars below F-2). This diapause can be terminated by long days relevant to the next spring season photoperiod (Ulf Norling, pers. comm.). In this experiment, we selected F-1 larvae for our analyses and, for the wintering cohort, corresponding changes in temperature and photoperiod, which are described below.

**Figure 1:**
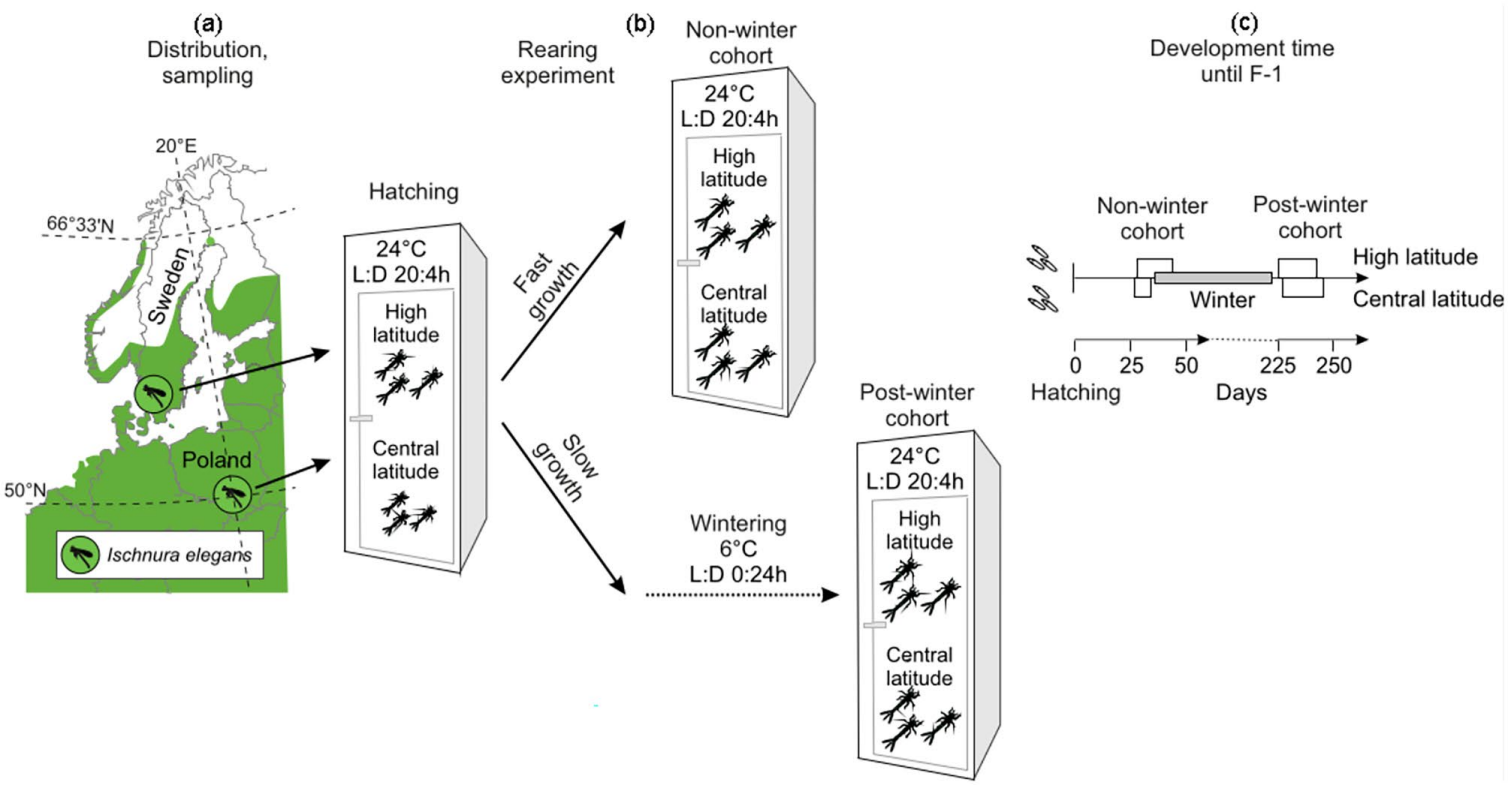
An overview of experimental design. (**a**) Map of central and northern Europe showing distribution of *I. elegans* (green area) and replicated high- (Sweden) and central (Poland) latitude sampling sites^26^. (**b**) A full-factorial experimental design where non- and post-wintering cohorts from high- and central latitudes were differentiated based on their growth rate and reared at 24 °C and photoperiod L:D 20:4 h, with the post-winter cohort experiencing wintering conditions, 6 °C and L:D 20:4 h. (**c**) Diagram showing larval development time and the entrance into pre-final instar (F-1; depicted by the white boxes) for the non- and post-wintering cohorts plus simulated wintering time period (grey box). Note that individuals entered wintering conditions at different dates as explained in the "Methods" section.

Mating pairs were collected from two southern Swedish (hereafter, high latitude) and two southern Polish (hereafter, central latitude) ponds between 22 and 23 June 2021 (Fig. 1, Supplementary Table S1). The ponds were situated in rural areas and had similar sizes and depths. The two Swedish and two Polish ponds were distant by 19.6 km and 67.9 km respectively, and were most likely not genetically isolated as gene flow is generally high in *I. elegans* across its native range^30,31^. Females were individually placed in plastic cups with perforated lids and wet filter paper for egg laying. Females were kept in a room at a temperature of 22 °C and natural daylight (photoperiod). In total, 40 clutches were used in the experiment, 10 per location.

### Experimental procedure: larval rearing

The experimental procedure is graphically shown in Fig. 1. The laboratory rearing part was designed to create two larval cohorts, non- and post-wintering, that were phenotyped (Analysis 1) and analysed for gene expression (Analysis 2). The distinction between the non- and post-winter cohort was made based on the larval growth rate, as in damselflies the cohort split generally arises due to asynchrony in the developmental timing and differences in the speed of growth^16,17^.

*Pre-winter conditions*. On 23 June 2021, upon arrival to the laboratory, egg clutches (hereafter, maternal lines) were kept in the climate incubator (Pol-Eko ST 700) with 22 °C and photoperiod of L:D 20:4 h. All larvae hatched between 07 and 13 July 2021. Once the eggs hatched, 10 individuals from each maternal line were randomly chosen and were placed in containers (size 17 × 12 cm, height 8 cm) filled with 700 mL of dechlorinated tap water and two nylon net strips, providing hiding space for larvae, and moved to another incubator with constant 24°C and L:D 20:4 h. The photoperiod corresponds to the longest day length at the high latitude collection site and favour development and growth rates in both studied latitudes^16^. This resulted with 2 latitudes × 2 ponds × 10 maternal lines = 40 containers × 10 larvae per container totalling 400 larvae at the beginning of the experiment. Throughout the experiment, individuals were raised in groups to mimic what happens in nature and were followed at the maternal line level, hence, non-winter and post-winter larvae from the same maternal line shared the same container at the beginning. We did not report the sex of each individual larva. However, a clutch contained the same proportion of males and females in *I. elegans*^32^ and we assumed that the final dataset was not sex-biased. Our field measurements (year 2021–2022) based on thermo-logger reads indicated that daily water temperature in shallow parts of the pond during the growth season can reach or be close to 24 °C in both latitudes (Supplementary Fig. S1). Hence, 24 °C is experienced in nature by *I. elegans* at both latitudes studied. For each container, the first one or two larvae (when two larvae moulted the same day) that entered F-1 were assigned to the non-winter cohort, they were phenotyped and preserved for gene expression analysis (*N* = 73). We assumed that individuals growing the fastest and reaching F-1 the first were less likely to overwinter at both latitudes compared to individuals at earlier instars and would emerge during the same growth season. The remaining larvae (*N* = 64) with slower growth rate and hence smaller sizes (below F-1 instars) were more likely to be directed for wintering and were assigned to the post-wintering cohort. Larvae were fed twice a day (week days) and once a day (weekend days) with laboratory-cultured *Artemia* nauplii (non-winter cohort).

*Wintering conditions.* For each container, once larvae for the non-winter cohort were phenotyped and preserved, the container with the remaining larvae directed for wintering were transferred successively into five different incubators with a weekly interval with decreasing temperature and photoperiod (hereafter, thermo-photoperiod) until they reached the winter conditions. Starting from 24°C and L:D 20:4 h, they were then successively moved to the incubators with the following conditions: 20°C and L:D 16:8 h; 16 °C and L:D 12:12 h; 12 °C and L:D 8:16 h; 8°C and L:D 0:24 h; 6 °C and L:D 0:24 h (winter conditions). Larvae from a post-wintering cohort were overwintered for 178 to 197 days. The variation in the length of the wintering period was caused by different dates of the entrance into F-1 for the non-wintering cohort across the different containers. Across containers, larvae entered F-1 between 4 and 23 August 2021 which caused the post-winter cohort to be transferred to the different incubators at different dates as well. From the date when thermo-photoperiod was reduced to 12°C and L:D 8:16 h and 8°C and L:D 0:24 h, larval feeding was reduced to, respectively, one portion of *Artemia* nauplii per day every day and to one portion of *Artemia* nauplii per day three times a week (Monday, Wednesday, and Friday) so that the wintering period in our experiment was not associated with a food stress.

*Post-winter conditions*. On 17 February 2022, we initiated spring conditions, and this date was fixed for all post-wintering cohorts. The small variation in the duration of the winter period had no influence of larval mass (Supplementary Fig. S2). Thermo-photoperiods were gradually increased with a two-day interval, from 6°C and L:D 0:24 h through 12 °C and L:D 8:16 h, 16 °C and L:D 12:12 h, 20 °C and L:D 16:8 h until 24 °C and L:D 20:4 h. As for the non-winter cohort, post-winter cohort was daily monitored for entering F-1. On the day larvae entered F-1, individuals were phenotyped and preserved for gene expression analysis (*N* = 48). The experiment ended when the last post-winter individual entered F-1. During this period larvae were fed twice a day.

### Response variables

To estimate larval survival, we counted individuals when the first larva per container from a non-winter cohort entered F-1 and when the last individual per container from post-winter cohort entered F-1 (i.e., end of the experiment). We could not attribute the cause of larval death, due to intrinsic, e.g. developmental errors, or extrinsic reasons, e.g. antagonistic interactions including cannibalism^33^, therefore the estimates should be interpreted with caution. When individuals for the non-winter cohort had started to enter F-1, the number of individuals in each container ranged from 0 to 6 totalling 137 individuals. We note that in one container all the larvae died before reaching F-1, and in two containers only one larva survived. When we initiated the spring conditions after wintering, the number of individuals per container ranged from 1 to 4. For both cohorts, every day before morning feeding, we checked for newly moulted F-1. Newly moulted individuals were placed in a separate incubator and were not fed, this fasting avoided the interference of feeding with mass and gene expression. At 14:00, larval wet mass (hereafter, mass) was measured with an electronic balance (Radwag AS.62). Then, larvae were photographed and head width and wing pad length were measured (Supplementary Table S2). Next, F-1 individuals were preserved in RNA Later and, after 24 h in the fridge, stored in -80 °C for gene expression analysis. We calculated larval development time as the number of days between hatching and entrance into F-1. For the post-winter cohort, the wintering period (between 178 and 197 days) was excluded from development time as during winter (temperature < 10 °C) larval growth is halted^34^. The growth rate between hatching and entrance into F-1 based on mass was calculated as mass / development time (GRM), and based on size as head width / development time (GRH).

### Gene expression

For central-latitude populations, we sequenced 22 individuals (11 non- and 11 post-winter individuals) and for high-latitudes populations, we sequenced 32 individuals (16 non- and 16 post-winter individuals) as survival was higher at high latitudes (see Results). Larvae selected for the gene expression analysis were at the same developmental stage but not exactly at the same age as they entered F-1 at different dates (Supplementary Table S2). On average, central-latitude individuals from the non-winter cohort reached the F-1 stage after 30 days and those from the post-winter cohort after 37 days (excluding the wintering period when larva growth stops). High-latitudes individuals from the non-winter cohort reached F-1 stage after 35 days and those from the post-winter cohort after 42 days.

*RNA extraction and sequencing*. For each larva, total RNA was extracted using RNAzol (MRC), and its integrity was assessed by agarose electrophoresis and on the Agilent 2100 Bioanalyzer. Libraries were prepared from up to 1 μg of the total RNA using NEBNext Ultra II Directional RNA Library Prep Kit Illumina (indexed with NEBNext Multiplex Oligos for Illumina (Dual Index Set 1 and Set2). The libraries were sequenced on DNBSEQ T7 (2 × 100 bp reads) at BGI. Sequencing generated between 20.3 and 44.5 million raw reads per individual. The non- and post-winter cohort were sequenced in two different runs, therefore, there is a potential batch effect. However, in our study, the non- and post-winter cohort libraries were prepared with the exact same kit and sequenced on the same equipment with the same parameters. It was demonstrated that, under such conditions, the batch effect is relatively small, if any^35,36^. The data are available from the Sequence Read Archive under accession PRJNA899331 (non-winter cohort) and PRJNA1040771 (post-winter cohort).

*Gene expression analysis*. Reads were mapped using hisat2 2.1.0^37^ on the *I. elegans* reference genome using the corresponding annotation generated by the Darwin Tree of Life Project (https://www.darwintreeoflife.org/; project ID: PRJEB46264)^38^. We excluded the sex chromosome to avoid any biases because we did not distinguish between males and females. The number of reads mapped on each gene was counted with featurecounts 2.0.3^39^, and only the uniquely mapped reads were kept. Differential gene expression analysis was performed using EdgeR v3.15^40^. We performed a gene expression analysis testing for the effects of wintering on gene expression for each latitude separately with a model including wintering (pre vs post winter) and ponds (2 ponds per latitude) as variables. Next, we compared the lists of differentially expressed genes (DEGs) between the two latitudes to distinguish between the genes differentially expressed in the same direction across the two latitudes (= shared response to wintering) and those specific of the latitude of origin (= specific response to wintering). We used Fisher’s exact test to test for a significant (non-random) overlap between the list of genes.

### Gene ontology

We created a custom Gene Ontology (GO) annotation as described in^28^. For each locus in the *I. elegans* genome, we retrieved the function and description from NCBI (https://www.ncbi.nlm.nih.gov/search/all/?term=ischnura%20elegans). Then, we extracted all genes described in insects with their respective GO terms from the UniProt database^41^ and compared this gene list with that of *I. elegans* genes. If a gene in *I. elegans* had a similar function and name and was associated with the exact same GO terms in at least three distinct insect species, we assumed the *I. elegans* gene to be involved in the same metabolic pathways. With this method, among the 21,087 genes described in the *I. elegans* genome annotation, we were able to assign GO terms to 4807 of them. We performed a GO term enrichment analysis with BiNGO v3.0.3 software^42^ with the most recent gene ontology annotation downloaded from The Gene Ontology Resource^43^. Gene ontology terms were considered significantly enriched if the FDR-adjusted *p*-value was < 0.05.

### Statistical analysis

All analyses were performed in R^44,45^. For univariate statistics, we used generalized linear mixed-effects models (GLMMs). We fitted the GLMMs using the function glmmTMB (glmmTMB package^46^). We included in the model the following predictors: latitude (central vs high latitude), wintering (non vs post winter) and their interaction as fixed factors and maternal lines nested within latitude as a random factor. For survival, we used the number of larvae per container as the response variable (‘Poisson’ distribution in glmmTMB). For the response variable mass, head width and wing pad, developmental time was added as a covariate as larvae reached F-1 at different ages. We ran separate model for each response variable: mass, head width, wing pad, GRH and GRM (‘Gaussian’ distribution in glmmTMB); the three variables head width, wing pad and GRH were log transformed to approach normal distribution. *P*-values were obtained using the Wald chi-square test (Wald X2) implemented in the car package^47^.

For multivariate statistics, we conducted a principal component analysis (PCA) based on the five phenotypic traits measured on the 121 individuals (*N* = 73 individuals for the non-winter and *N* = 48 individuals for the post-winter cohort). To account for the fact that damselflies were of different ages, for mass, head width, and wing pad, we ran a linear model with developmental time as covariate and extracted the residuals that were subsequently used for the PCA. All variables were scaled and centred before running the PCA. We also ran a multivariate analysis of variance (MANOVA) to test for a significant effect of latitude, wintering, and their interaction on the combination of the five phenotypic traits. In a similar way, we also ran a PCA with gene expression data of the 54 individuals sequenced (see ‘Gene expression’ section) and we used permutational multivariate analysis of variance (PERMANOVA) to test for overall differentiation among the transcriptomic profiles with regard to latitude, wintering, and their interaction (adonis2 function, vegan package^48^, number of permutations = 10,000).

## Results

### Effects of wintering on the phenotype

In total, 137 individuals survived when the first larva per container had started to enter F-1 with no difference observed between the two latitudes (Wald X^2^ = 2.62, Df = 1, p = 0.106). After we collected the 73 individuals for phenotyping and gene expression analysis, we initiated winter conditions with 64 individuals and 48 survived at the end of the experiment. The comparison of these two groups of individuals revealed no significant effect of wintering on survival (Wald X^2^ = 2.23, Df = 1, *p* = 0.135) but a significant effect of latitude (Wald X^2^ = 7.11, Df = 1, *p* = 0.008) with the lower survival in central than high latitude at the end of experiment (N _central_ = 14; N _high_ = 34) (Supplementary Fig. S3).

**Figure 2:**
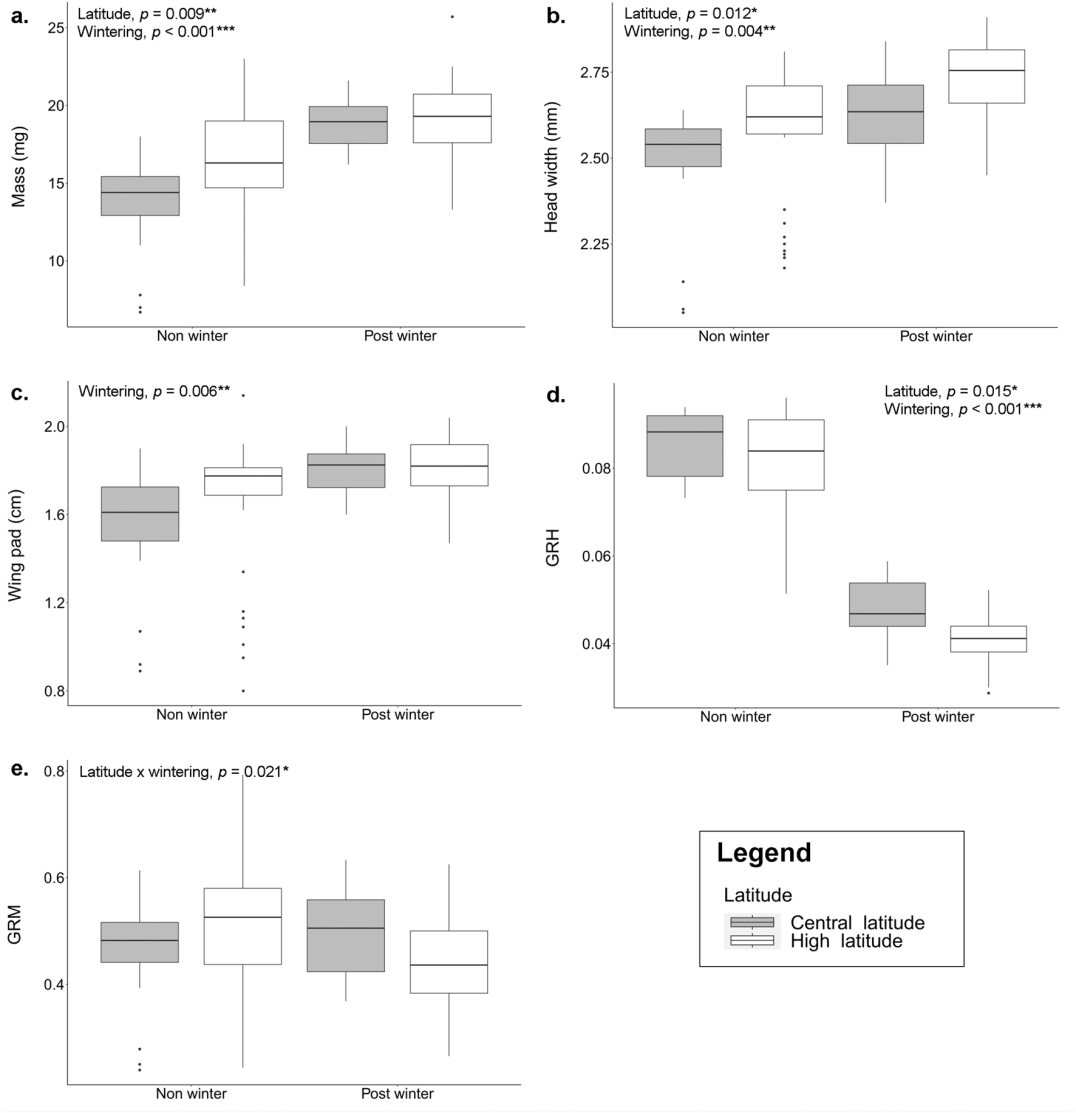
Boxplots showing the effects of wintering and latitude on (**a**) mass, (**b**) head width, (**c**) wing pad, (**d**) growth rate based on head width (GRH) and (**e**) growth rate based on mass (GRM). For each panel is indicated the significance of each variable tested by GLMM (Table 2).

First, we ran distinct models for each variable, results are presented in Table 1. Univariate statistics showed a clear effect of wintering on all variables especially on GRH. For the non-winter cohort, larvae had lower mass, lower values for head width and wing pad, and higher values for GRH and GRM compared to F-1 larvae from post-winter cohort (Fig. 2). Latitude had significant effects on mass, head width, and GRH with higher values for mass and head width and lower values for GRH at high- than central latitude. The interaction latitude × wintering was significant only for GRM (Fig. 2e).

**Table 1:** Effect of developmental time (Dev. time; covariate), latitude, wintering, and the interaction latitude × wintering on the five phenotypic traits: mass, head width, wing pad, growth rate based on head width (GRH), and growth rate based on mass (GRM).

		Mass	Head width	Wing pad	GRH	GRM
	Df	*p(F)*	*p(F)*	*p(F)*	*p(F)*	*p(F)*
Dev. time	1	0.990 (0.00)	0.225 (1.47)	0.692 (0.16)	–	–
Latitude	1	0.009 (6.85) **	0.012 (6.26) *	0.180 (1.80)	0.015 (5.88) *	0.924 (0.01)
Wintering	1	< 0.001 (18.3) ***	0.004 (8.21) **	0.006 (7.63) **	< 0.001 (934) ***	0.058 (3.60)
Latitude × wintering	1	0.085 (2.96)	0.568 (0.33)	0.256 (1.29)	0.136 (2.22)	0.021 (5.33) *

Table shows degree of freedom (Df), *p*-value and *F*-value in parentheses. Significance is indicated in bold: *** *p *< 0.001, ** *p * < 0.01, * *p *< 0.05.

**Figure 3:**
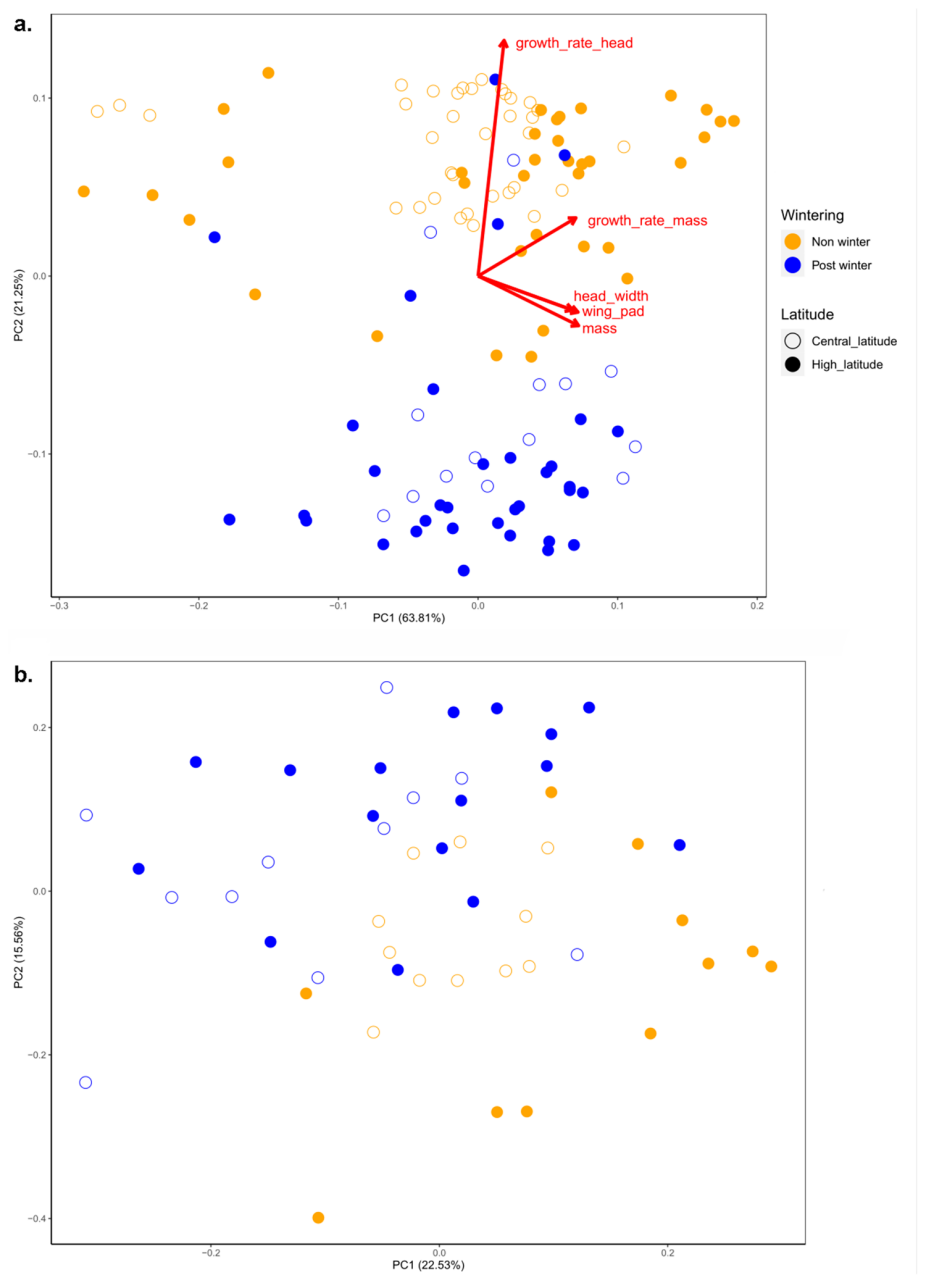
(**a**) Principal component analysis (PCA) plots based on the five phenotypic traits: mass, head width, wing pad, growth rate based on head width (GRH) and on mass (GRM) measured on 121 individuals. (**b**) PCA plot showing gene expression differences between the 54 individuals sequenced. Colours indicate the non- (yellow) and post-winter (blue) cohort and shapes indicate the latitude of origin (open circle = central latitude; filled circle = high latitude).

Next, PCA plots revealed two distinct clusters corresponding to the non- and post-winter cohort separated along the PC2 axis and differing mainly in GRH with higher values in individual reaching F-1 before winter (Fig. 3a). We used MANOVA to test for the effect of wintering, latitude, and their interactions on the combination of the five phenotypic traits. We found an overall significant difference in this combination of traits between latitude and between the non- and post-winter cohort. But we did not find latitudinal-specific differences in response to wintering as shown by the non-significant interaction latitude × wintering (Table 2a).

**Table 2:** Results of (a) the MANOVA of the five phenotypic traits: mass, head width, wing pad, growth rate based on head width (GRH) and growth rate based on mass (GRM) and (b) the permutational analysis of variance (PERMANOVA) of gene expression, testing for the effect of latitude, wintering and the interaction latitude × wintering.

a. Phenotype	DF _num/den_	Approx. F	*p*
Latitude	5/111	37.4	< 0.001 ***
Wintering	5/111	279	< 0.001 ***
Latitude × wintering	5/111	2.14	0.066

b. Gene expression	R^2^	*F*	*p*
Latitude	0.04	1.98	0.059
Wintering	0.05	2.70	0.015 *
Latitude × wintering	0.02	1.18	0.291

Table shows for (a) numerator and denominator degree of freedom (DF _num/den_), approximation of *F*-value (approx. F) and *p*-value, and for (b) percentage of variance explained (R^2^), *F* value and *p*-value. Significance is indicated in bold: *** *p* < 0.001, * *p* < 0.05.

### Effects of wintering on the transcriptome between central and high latitudes

PCA and PERMANOVA test showed significant differences between the non- and post-winter cohort at the whole-transcriptomic level (Df = 1; *F*-value = 2.70; *p* = 0.016) whereas the effects of latitude and of the interaction latitude × wintering were not significant (Fig. 3b, Table 2b).

**Figure 4:**
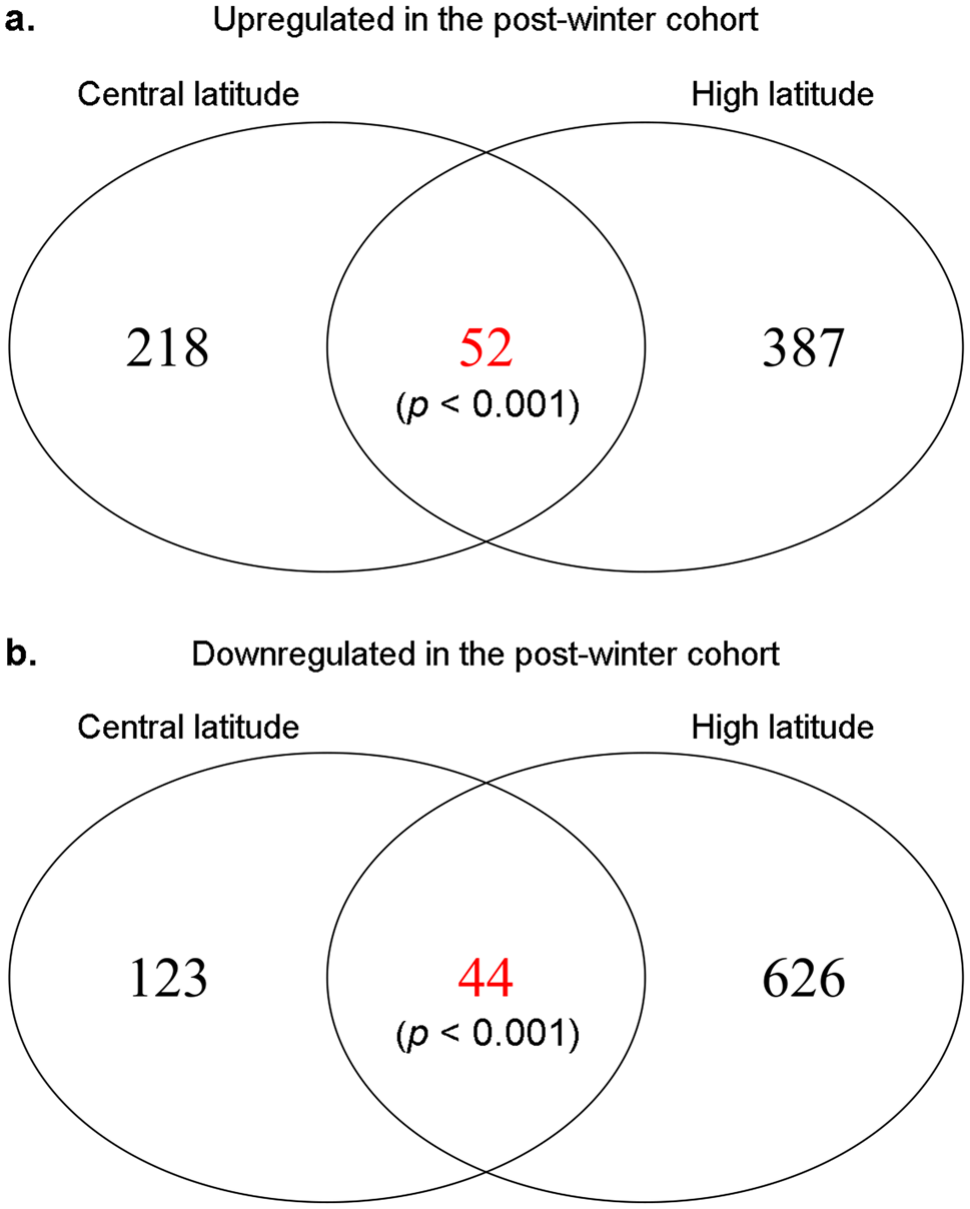
Venn diagram showing the number of genes differentially expressed between the non- and post-winter cohort for each latitude and their overlap. Fisher’s exact test was used to test for a significant (non-random) overlap and p-values are indicated in parentheses.

When looking at each latitude separately, we found 437 differentially expressed genes (DEGs) between the non-winter and post-winter cohort at central latitudes with 270 genes upregulated and 167 downregulated in the post-winter cohort. At high latitudes, we found 1109 DEGs with 439 upregulated and 670 downregulated in the post-winter cohort. Of those, 52 genes were upregulated and 44 downregulated at both latitudes (Fig. 4). The overlap in gene expression between the up- and downregulated genes across the two latitudes was greater than expected by chance as tested by Fisher’s exact test indicating significant similarities in the effects of wintering at the gene expression level (up-regulated genes: observed overlap = 52; expected overlap = 10.6; *p* < 0.001. Down-regulated genes: observed overlap = 44; expected overlap = 10.0; *p* < 0.001).

### Shared response to wintering

In total, 52 genes were upregulated in the post-winter cohort at both latitudes. Among those, 33 were known and only 17 had associated GO terms (Supplementary Table S3). Four genes were involved in structural integrity, morphogenesis, and cytoskeleton and four genes in sensory system (olfactory and visual perception). Two genes were related to growth regulation and morphogenesis. The remaining genes had various functions such as calcium binding, transferase, deaminase, ion channel, protein oligomeric formation, signal transduction or transporter (L-glutamate, lipid).

Among the 44 that were downregulated at both latitudes, 26 were known and only six had associated GO terms. Two of them are transporters (ABC transporter and L-ascorbate:sodium symporter activity), two are calcium binding genes and one is also involved in structural integrity, the last two genes had various functions associated with membrane or protease activity.

### Specific response to wintering

Among the 437 DEGs between the non-winter and post-winter cohort at central latitudes: 218 genes were up-regulated and 123 downregulated in the post-winter cohort and were specific to central-latitude individuals (Supplementary Table S4). Gene ontology (GO) term enrichment on the 218 DEGs up-regulated post wintering revealed similar GO terms associated with cytoskeleton such as cilium, cell projection or axoneme for biological process and cellular component; and to serine activity for molecular activity. For the genes downregulated post wintering, DEGs were related to in carbohydrate transport for biological process; to membranes for biological process; and to proteolysis activity for molecular activity.

Among the 1109 DEGs between the non-winter and post-winter cohort at high latitudes: 387 genes were up-regulated and 626 downregulated in the post-winter cohort and were specific to high-latitude individuals (Supplementary Table S5). Gene ontology (GO) term enrichment on the 387 DEGs up-regulated post wintering revealed GO terms related to cell communication, signal transduction and synaptic signalling for biological process; to membrane and synaptic membrane for cellular component; and to gating and neurotransmission for molecular activity. For the genes downregulated, GO terms were related to electron transport and respiration for biological process; to mitochondria for cellular component; and to cuticle and ribosome activity for molecular activity.

## Discussion

In the present study, we investigated phenotypic and transcriptomic differences between a non- and post-winter cohort across two latitudes in a facultative-diapausing damselfly, *I. elegans*. We demonstrated clear differences between the non- and post-winter cohort at the phenotypic level, with the post-winter cohort damselflies being generally larger and heavier than the non-winter cohort, and these effects were shared across the two latitudes for most of the life-history traits. At the transcriptomic level, results showed a strong effect of wintering on gene expression and a small fraction overlapped across latitudes especially for genes related to morphogenesis.

### Effects of diapause on the phenotype

We found significant effects of winter diapause on all the life-history traits we measured; the post-winter cohort being generally larger and heavier than the non-winter cohort and showing slower growth rate. This indicates that accelerated development of non-winter cohort was (at least partially) caused by stronger seasonal time constraint comparing to overwintering cohort, the later possibly going through additional moult and eventually gaining larger size and heavier mass^16^. It has been suggested that winter diapause might bear costs, as diapause may be associated with other stressful factors such as low temperature, limited resources available and reduced food intake^49^. Indeed, food availability and nutrients reserves are critical component of the diapause process which may affect survival^6,20^. In our study, we did not find greater mortality after the simulated winter conditions neither potential developmental lags nor delays as larvae developed relatively fast to the penultimate stage in the post-winter cohort. This was probably due to the fact that, in our experiment, larvae were fed during the winter period (though, with a lower amount of food). In addition, rearing individuals in group may also increase the growth rate and decrease developmental time as these two parameters may be density-dependent and may also vary between sexes that were not reported here^50,51^. This was consistent with previous studies where the authors gave the same amount of food to diapausing and non-diapausing individuals, and observed no difference in survival in bruchid beetle^10^. However, we found differences in survival between the two latitudes. This may be explained by lower survival of fast-growing and more time constrained individuals, as shown in earlier studies^52^, by variable cannibalistic behaviour depending on the latitude of origin or by variable predation risk depending on the density of larvae per container which may cause additional stress, as noted in previous studies^51,53^.

Apart from survival, our results aligned with previous findings in *I. elegans*, where faster growth rate and reduced development time, as found in the non-winter cohort or when density of individual increases, often come at a cost of having a smaller mass^28,33,51^. However, previous studies tended to observe the opposite patterns with a smaller size in diapausing individuals^5,18,19^. It is noteworthy that these studies were conducted on holometabolous insects with different developmental stages compared to hemimetabolous insects (including *I. elegans*). Some aspects of diapause may potentially differ between these two modes of development. Another explanation may arise from the timing of removal of experimental larvae. Indeed, during diapause, there are two periods associated with an increase in food intake and metabolic rate: the preparation of diapause to accumulate reserves, i.e. when photoperiod and temperature decreases, and during the resumption period which generally occur after diapause when metabolic rate increases in order to finish larval development and subsequently metamorphose^1,49,54^. In our study, for the post-winter cohort, larvae were collected between one and three weeks after diapause. Larvae may have probably increased their food intake due to high metabolic demands associated with the resumption period. This may have probably exacerbated the difference in mass and size with the non-winter cohort that experienced only summer conditions.

Our results also showed that the effects of wintering on life-history was mostly independent of the latitude, as none of our variables, except GRM, exhibited significant latitude × wintering interaction. The two latitudes differed significantly in all traits measured with faster growth and lower mass in central-latitude individuals compared to high-latitude individuals, in line with previous studies^15,28^. In general, the differences observed between latitudes in the non-winter cohort remained after winter diapause. In previous studies, some differences were observed in duration of diapause along a latitudinal gradient in blow fly larvae^19,21^ and in odonates^55^, indicating that other aspects of diapause may exhibit constitutive genetic differences at the species level. Hence, latitudinal differences seemed to be more related to the control and incidence of diapause either through plasticity, i.e. induced by different temperatures and photoperiods between latitudes, or constitutive genetic differences, rather than to its direct effects on the phenotype.

In general, variations in life-history traits are expected in species with variable voltinism, and such variations may be manifested along environmental gradients but also within a population or a maternal line leading to different cohorts. In our study, we did not find clear costs of winter diapause on fitness components (i.e., survival) or on life-history traits between or within latitudes. In that sense, facultative diapause which is generally due to asynchronous development, may be seen as a risk-spreading strategy arising from environmental uncertainties^7,56^. Indeed, diversifying the response to the environment and spreading larval development across several years, with individuals developing faster and others going for diapause and emerging in the next seasons, increase the probabilities to have progeny that develop and reproduce under favourable conditions.

### Effects of diapause on the transcriptome

We explored transcriptomic differences in the non- and post-winter cohort between the two latitudes. We found that most of the genes differentially expressed between the non- and post-winter cohort were specific for the latitude of origin but a significant fraction overlapped across the two latitudes. Diapause has evolved independently many times during insect evolution and comparative studies showed similarities in gene expression in the response to diapause across different insect taxa and other ectotherms suggesting a shared transcriptomic basis and evolutionary convergence underpinning diapause (‘shared genetic toolkit’^57,58^). For instance, a shared transcriptomic basis in response to diapause was found between *Drosophila melanogaster* and *Caenorhabditis elegans*^59^ or between Diptera species^60^. In general, the genes part of the shared response were more likely to be associated with stress (starvation), developmental processes and morphogenesis, translation, cellular structure, basic metabolism and energy consumption which include lipids, carbohydrates, and amino acids metabolism^54,58^.

In our study, among the shared fraction of genes, most of them were related to morphogenesis, growth regulation, and cell structure (cytoskeleton) and tended to be up-regulated in the post-winter cohort. This was consistent with previous findings cited above and with the differences in growth and development between the two cohorts. Interestingly, previous studies pointed to an important role of the sensory system, the visual system in particular, in initiating or terminating diapause^12,61^. Indeed, insects rely on photoperiod and probably use visual cues to initiate their development after winter. In line with these findings, we identified two genes related to sensory system and vision (*NEITHER INACTIVATION NOR AFTERPOTENTIAL PROTEIN C* and *GUANINE NUCLEOTIDE-BINDING PROTEIN SUBUNIT BETA-2*) that were both up-regulated in the post-winter cohort. The remaining genes up- or down-regulated had general functions such as transporter or signal transduction, these genes are ubiquitous and are probably related to general metabolic functions.

Then, when looking at the genes specific of central-latitude populations, we found that most of them were involved in cellular organization and carbohydrate metabolism, and may have a role in developmental process and energy consumption. The analysis also revealed different members of the serine family with putative functions in immune response and phenoloxidase activation and were previously shown to be associated with nutritional status and aging^62^.

Among the genes specific of high-latitude populations, we found genes related to cell communication, signal transduction, synaptic signalling, and neurotransmission. The nervous system, in particular the circadian clock, is tightly linked with integration of environmental cues which is an important component of diapause^57,61^. Although we found GO terms related to the nervous system in line with a previous study that showed genetic differentiation for such genes along a latitudinal gradient^31^, we did not find enriched GO terms directly associated with the circadian clock, at least for the ones we could define with the current knowledge. Other genes were located in mitochondria and related to respiration and energy production suggesting that, in our study, energy metabolic pathways tended to be part of the specific rather than the shared response to diapause.

Finally, central- and high-latitude populations differ in voltinism and life-history strategies with a faster development and smaller size and mass at central latitudes. Previous genomic studies conducted on *I. elegans* demonstrated genetic differentiation between central- and high-latitude populations^31^ and constitutive genetic differences with regard to growth rate along a latitudinal gradient^30^. In addition, central- and high-latitude populations may also differ in their plastic responses to environmental cues (i.e. temperature and photoperiod) to initiate or terminate diapause as previously demonstrated in other insect species^19,21^. Altogether, this may contribute to the specific response to diapause observed at the transcriptomic level.

## Conclusion

In conclusion, our comparison of a non- and post-winter cohort in a damselfly with variable voltinism revealed important differences in growth and development without clear costs in terms of reduced larval survival or developmental lags in the post-winter cohort, yet, these effects were rather similar across the two latitudes. However, we focused only on the larval stage and to what extent our observations on the non- and post-winter cohort manifest at the adult stage and especially on adult fecundity requires further investigations. Finally, our results supported the idea of a shared transcriptomic basis (‘shared genetic toolkit’) underpinning diapause at the intraspecific level between central- and high-latitude populations.

### Supplementary Information

Supplementary Information 1.

Supplementary Information 2.

Supplementary Information 3.

Supplementary Information 4.

Supplementary Information 5.

## Data Availability

Phenotypic data generated and analysed during this study are included in this article (and its Supplementary Information files). Transcriptomic data are available from the Sequence Read Archive under accession PRJNA899331 (non-winter cohort) and PRJNA1040771 (post-winter cohort).
