# Supplementary material for: Effects of overwintering on the transcriptome and fitness traits in a damselfly with variable voltinism across two latitudes
Source: Sci Rep. 2024 May 28;14:12192. doi: 10.1038/s41598-024-63066-z (PMC11133422; doi:10.1038/s41598-024-63066-z)
Supplement: Supplementary file 1 — Supplementary Information 1. [file 41598_2024_63066_MOESM1_ESM.docx]

Supplementary information

**Effects of overwintering on the transcriptome and fitness traits in a damselfly with variable voltinism across two latitudes**

Guillaume Wos^1^, Gemma Palomar^1,2^, Maria J. Golab^1^, Marzena Marszałek^3^, Szymon Sniegula^1^

^1^Institute of Nature Conservation Polish Academy of Sciences, al. Adama Mickiewicza 33, 31-120 Krakow, Poland

^2^Department of Genetics, Physiology and Microbiology, Faculty of Biological Sciences, Complutense University of Madrid, José Antonio Novais, 12, 28040 Madrid, Spain

^3^Institute of Environmental Sciences, Jagiellonian University, Gronostajowa 7, 30-387 Kraków, Poland

**Fig. S1**. a) Monthly and b) weekly temperatures for each Swedish (SW; high latitude, broken line) and Polish (PL; central latitude, solid line) pond measured using dataloggers. Dataloggers were installed) in each pond (50 cm depth) for one year or only several months for the year 2021 or 2022.


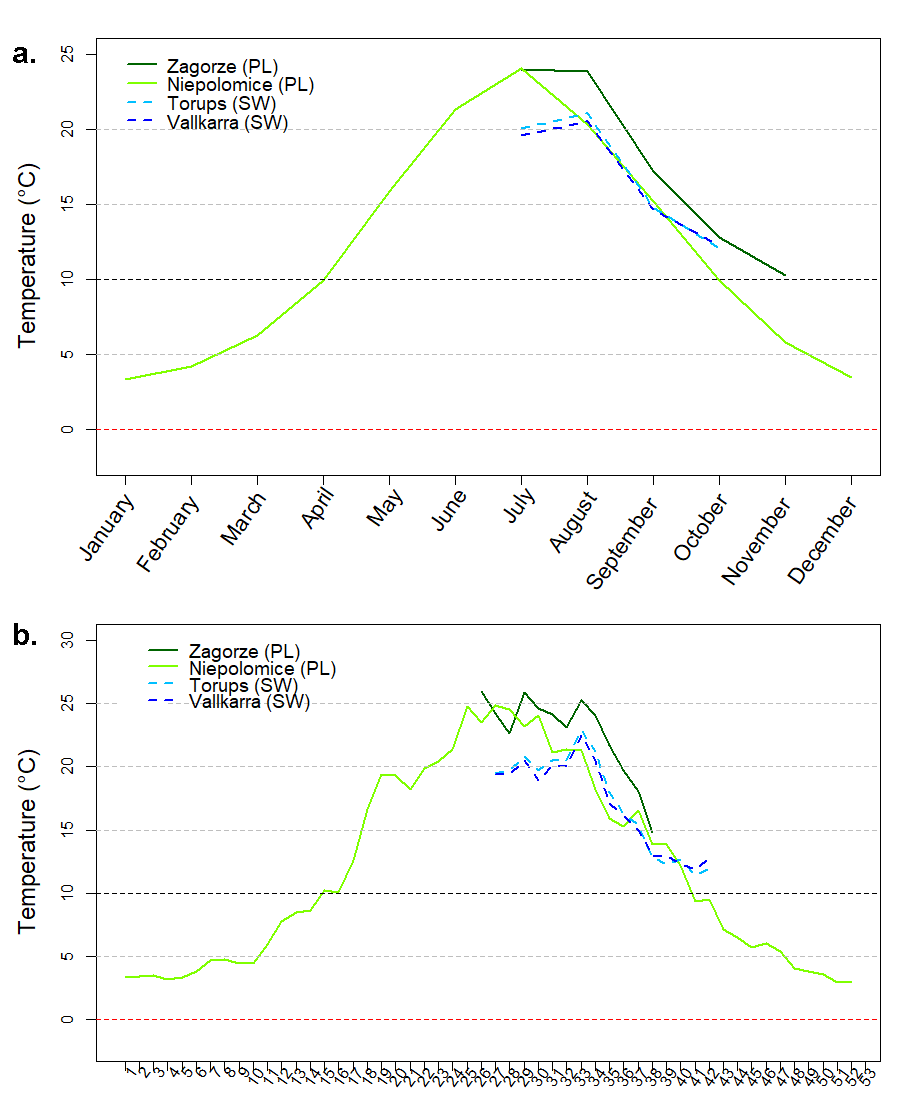


**Fig. S2**. Correlation between mass of the post-winter cohort at F-1 stage and the time spent in winter conditions that ranges between 178 and 197 days for a) central and b) high latitude.


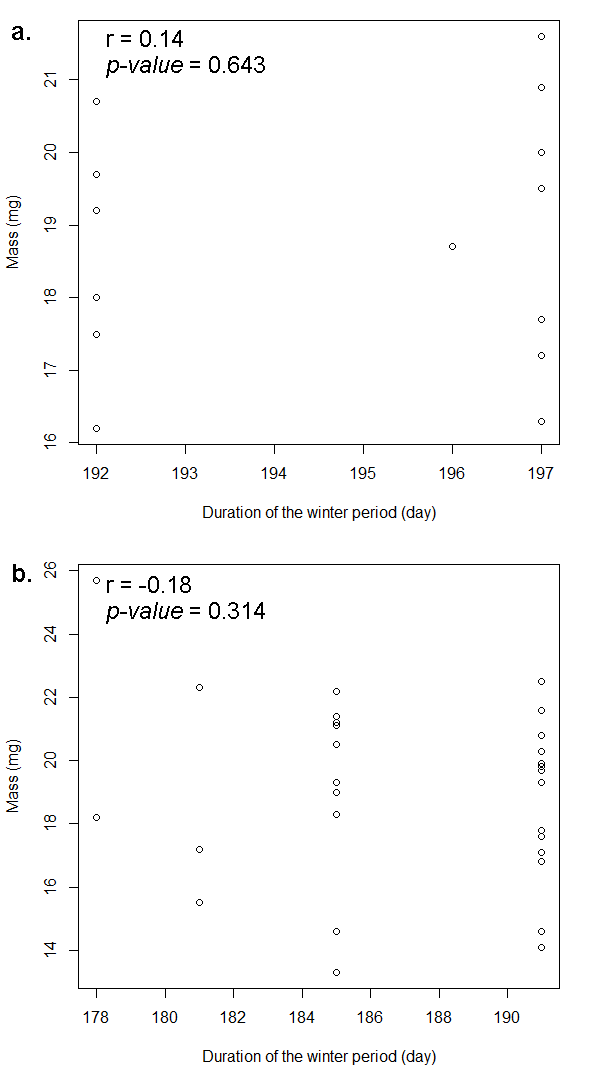


**Fig. S3**. Boxplot of the number of individuals per container for central and high latitude (20 containers per latitude) in the non- (*N* = 64) and post-winter (*N* = 48) cohort.


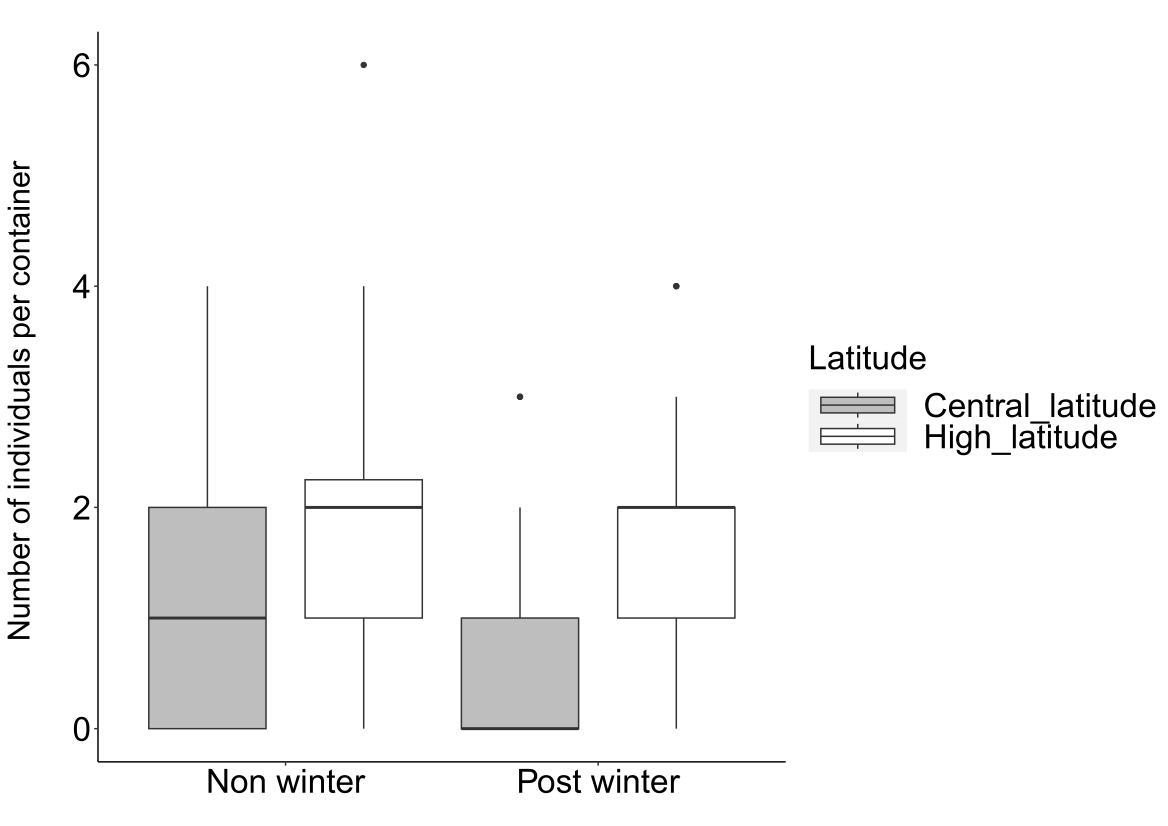


**Table S1**. Sample size in each experimental group at the end of the experiment.

|  |  | **Coordinates** | |  |  |
| --- | --- | --- | --- | --- | --- |
| **Latitude** | **Location** | **Latitude** | **Longitude** | **Cohort** | **N** |
| High latitude | Vallkara | 55.738166 | 13.153274 | Non-winter | 18 |
| High latitude | Torups | 55.564293 | 13.205614 | Non-winter | 19 |
| Central latitude | Niepolomice | 50.10875 | 20.348707 | Non-winter | 19 |
| Central latitude | Zagorze | 50.083352 | 19.39736 | Non-winter | 17 |
| High latitude | Vallkara | 55.738166 | 13.153274 | Post-winter | 15 |
| High latitude | Torups | 55.564293 | 13.205614 | Post-winter | 19 |
| Central latitude | Niepolomice | 50.10875 | 20.348707 | Post-winter | 6 |
| Central latitude | Zagorze | 50.083352 | 19.39736 | Post-winter | 8 |
|  |  |  |  |  | Total = 121 |
